# Colorectal Cancer Chemoprevention: Is This the Future of Colorectal Cancer Prevention?

**DOI:** 10.1100/2012/327341

**Published:** 2012-04-29

**Authors:** A. Manzano, P. Pérez-Segura

**Affiliations:** Oncology Department, Hospital Clínico San Carlos, 28040 Madrid, Spain

## Abstract

Colorectal cancer (CRC) is presently one of the most common causes of cancer-related death in our setting and affects a great number of people each year. Screening strategies are commonly used but they do not seem enough to avoid CRC development or prevent completely its mortality. Because of this fact other prevention strategies have gained interest in recent years. Chemoprevention seems to be an attractive option in this setting and several drugs have been studied in this field. This review is focused on salicylates, nonsteroidal anti-inflammatory drugs (NSAIDs) and cycloxygenase-2 inhibitors (COXIBs), whose mechanism of action could be directly related to colon cancer chemoprevention.

## 1. Background

Colorectal cancer (CRC) is the second leading cause of death in the U.S. and affects approximately one million people each year, with a 5-year survival rate of 62% [1]. Approximately 20% of the cases of CRC have familial aggregation with more than two first-degree family members affected, whereas 5–10% occur in the context of a hereditary syndrome [2].

The development of colorectal cancer is a complex process involving multiple molecular pathways, since the formation of adenomas to the development of carcinoma in the digestive tract (the so-called “adenoma-carcinoma sequence”), in a process that can last several decades [3]. Thus adenomas are considered a surrogate variable for the development of CRC in clinical trials.

Although screening strategies (blood in stool, endoscopic and CT-colonoscopy) have supposed a great advance in the early detection of these tumours, they are associated with inconveniences such as their cost and associated morbidity. Moreover screening does not necessarily prevent the development of cancer or prevent mortality. Therefore, interest in primary prevention research has increased in recent years. In this regard, multiple attempts to modify lifestyle and dietary factors to try to reduce the incidence of cancer have been promoted. However, some studies, many of them observational or case-control, have yielded conflicting data [3]. Consequently, in the past 20 years, chemoprevention studies have grown in importance.

Cancer chemoprevention is defined as the use of chemical agents in healthy individuals to block, reverse, or delay the development of invasive cancer. Although several drugs have been studied, this review focuses on salicylates and nonsteroidal anti-inflammatory drugs (NSAIDs), as they constitute a group of agents whose mechanism of action could be directly related to colon cancer chemoprevention. Both treatment groups are inhibitors of the cyclooxygenase (COX) enzyme responsible for the transformation of arachidonic acid to prostaglandins, which are involved in cell proliferation and apoptosis [4].

**Figure 1:**
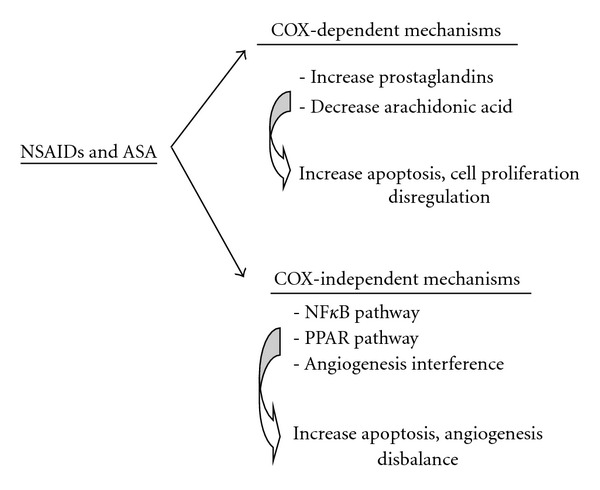
Scheme of COX-dependent and-independent mechanisms related with NSAID and ASA.

In humans, three isoforms of the COX enzyme exist. COX1 is expressed constitutively in all tissues and is involved in maintaining the integrity of the gastric mucosa and platelet aggregation, among other functions. The function of COX3 remains to be determined, whereas the expression of COX2 is inducible in both inflammatory processes and tumorigenesis. Thus, COX2 is overexpressed in colon tumours and adenomas, with expression not observed in normal gastrointestinal mucosa. The mechanism of action of salicylates and NSAIDs as chemopreventive agents are not fully understood; however it is postulated that they act in both COX-dependent and -independent mechanisms (Figure 1). In COX-dependent mechanisms, COX inhibition produces a decrease in the levels of prostaglandins and their derivatives (prostacyclins and thromboxane), producing a decrease in the processes involved in cell proliferation. Conversely, COX-dependent mechanisms also lead to an increase in the level of arachidonic acid, which promotes apoptosis. Furthermore, experimental studies have shown that NSAIDs induce apoptosis in tissues that do not express the COX enzyme. These COX-independent mechanisms are still being studied and may involve different pathways like NF*κ*B, the peroxisome-*γ* proliferator-activated receptor (PPAR) and its ligands and could also interfere with angiogenesis [4, 5]. 

This review will address the scientific evidence in randomised clinical trials for these agents in both sporadic and hereditary CRC chemoprevention fields.

## 2. Sporadic Colorectal Cancer

Sporadic CRC represents 75% of the total CRC cases and is the third most common cancer in the world and the second leading cause of cancer death [6]. Adenoma is the precursor lesion to CRC in a sequence that may last 10–15 years.

### 2.1. Aspirin (ASA)

The role of ASA in chemoprevention was first provided by Kune et al. in a case-control study in 1988, where an odds ratio (OR) of 0.53 for CRC incidence was observed for chronic users of aspirin compared with nonusers [7]. Since the publication of this report, other epidemiological observational studies have shown similar results regarding the incidence of CRC and the development of adenomas.

In response to these studies, randomised trials were designed to evaluate the role of aspirin in preventing cancer. Two of the studies determined the incidence of CRC in large healthy populations and were unable to demonstrate a chemopreventive effect for ASA. The first was the Physicians' Health Study (PHS), a blind, randomised study designed to test the effect of low doses of aspirin on the incidence of cardiovascular events and CRC [8]. In 1993, Gann et al. published the results of CRC incidence after 5 years of follow-up [9]. In this study, 22071 healthy men were randomised to receive either placebo or 325 mg of aspirin every other day. The primary endpoints of incidence of invasive CRC (RR 1.15, 95% CI 0.80–1.65) and the incidence of adenomas (RR 0.85, 95% CI 0.68–1.10) were both negative. In 1998, Stürmer et al. published an update of these data over a 12-year period, demonstrating a lack of effect with an RR of 1.03 (95% CI 0.83–1.28) for CRC incidence [10].

The second study, by Cook et al. was published in 2005 [11]. In this study, 39876 healthy women were randomised to receive placebo or 100 mg aspirin every other day. The endpoints of the study were to establish (1) the incidence of invasive cancer in any location and (2) the incidence of breast cancer, CRC, and lung cancer. The study was unable to demonstrate an effect, with an RR of 1.01 for any cancer and an RR of 0.97 (95% CI 0.77–1.24, *P* = 0.08) for CRC incidence.

In 2003, under the assumption that the dose used in these two studies was not optimal to test the power of ASA in chemoprevention, three studies were simultaneously published from selected populations (with the use of colonoscopy at baseline and follow-up). The main objective was to correlate the effect of different doses of ASA with the incidence of adenomas. The first study by Baron et al. explored the effects of two doses of aspirin (81 mg or 325 mg twice daily) versus placebo in 1121 patients with a recent history of adenomas [12]. The study was positive for the primary endpoint (detection of one or more adenomas at first colonoscopy) at the 81 mg dose (38% versus 47% in the placebo arm, *P* = 0.04). Moreover, the RR for the incidence of adenomas was 0.81 (95% CI 0.69–0.96) with a 40% reduced risk for advanced lesions at the 81 mg dose. There was no statiscally significant difference in the risk of death or risk of bleeding compared with placebo.

In the second study, APPAC study, 272 patients with a personal history of colorectal adenomas were randomised to receive 160 mg or 300 mg of lysine acetylsalicylate or placebo for 4 years [13]. The study was not designed to study the differences between the two doses of salicylates and was negative at one year of follow-up with an RR of 0.73 (95% CI 0.52–1.04, *P* = 0.08) for the incidence of adenomas.

The third study, by Sandler et al., was published in 2003. In this trial, 635 patients received placebo or 325 mg ASA daily. It was closed prematurely after the first interim analysis, as the main objective was achieved. The incidence of 1 or more adenomas was 17 versus 27% (*P* = 0.004) with an RR of 0.65 [14]. No significant differences in the size of polyps or incidence of advanced adenomas (larger than 1 cm or the presence of villous component) were found. The population in this study included patients with a personal history of CRC (Dukes A, B and C), a high-risk population that may partially explain these results (Table 1).

Subsequently, a meta-analysis of four studies (the three published in 2003 with an additional one published in 2008 [15] and positive for ASA) was published in 2009 [16]. In this study, with a population of 2698 and a median follow-up of 33 months, the RR for the incidence of adenomas was 0.83 (95% CI 0.72–0.96), and the incidence of advanced lesions was 0.72 (95% CI from 0.57 to 0.90). The number of CRCs diagnosed was low, with no statistically significant difference compared with the placebo arm. Although the comparison between the low dose (450 patients) and the high dose (1228 patients) of aspirin appeared to show a greater effect for the lower doses, these data should be interpreted with caution due to the small number of trials included in the meta-analysis. Serious side effects were infrequent, and no difference in terms of gastrointestinal toxicity was noted. However, 12 individuals suffered from a stroke, all in the ASA group (*P* = 0.02). 

The heterogeneity in terms of selection criteria, study population as well as dose and duration of treatment in the previous studies made it difficult to draw conclusions on the clinical use of ASA. The U.S. Preventive Services Task Force (USPSTF) conducted a meta-analysis, published in 2007, that included randomised trials, case-control, and cohort studies [17]. This meta-analysis established an RR for adenoma incidence of 0.82 (95% CI 0.7–0.95) and a decrease in CRC incidence of 22% (for the cohort studies). Mortality data were limited and inconsistent; thus, conclusions were not made. In terms of toxicity, the use of aspirin was associated with an increased risk in the incidence of gastrointestinal bleeding with an RR 1.5–3, which was dose dependent (higher for higher doses). In the analysis of the data, the benefit of aspirin was greater with higher doses compared with those used for cardiovascular prevention. Furthermore, the benefit was higher for the high-risk CRC population (family history) and for prolonged treatment.

Subsequent to this meta-analysis, few additional studies have been published. Only two studies by by Flossman [18] and Rothwell [19] provide data on prolonged treatment. Both studies, with 20-year follow-up in a population cohort from cardiovascular prevention studies (healthy population with no history of previous adenomas), studied CRC incidence as the primary endpoint. With 7588 and 14033 patients enrolled, respectively, and with different doses of aspirin (30 mg–1200 mg) for a median of 5-6 years, both studies demonstrate a benefit in CRC incidence with a similar HR (0.74 and 0.76, resp.). When the analysis was separated by intervals of 10 years, the benefit of ASA occurred mainly in the second decade. The study by Rothwell et al. also demonstrated a reduction in CRC mortality with an HR of 0.65 (0.48 to 0.88, *P* = 0.005). However, none of the initial studies were designed to study CCR incidence, as they were cardiovascular preventive studies conducted prior to the colonoscopy screening era. Thus, these data should be interpreted with caution. 

Based on the data published to date, we can conclude that aspirin is effective in reducing adenomas and CRC incidence (modestly) and that its benefit is higher in high-risk populations, with an acceptable safety profile excluding risk populations for bleeding. It remains to be determined what the optimal dose and duration of treatment are and whether this strategy is associated with a reduction in CRC mortality with an acceptable toxicity profile for healthy individuals.

### 2.2. Cyclooxygenase-2 Inhibitors (COXIBs)

The selective inhibition of the COX2 isoform by COXIBs makes these drugs attractive for preventive studies to minimise side effects, such as the gastrointestinal toxicity observed with COX1 inhibition.

After data from approximately 40 observational studies demonstrated the utility of COXIBs as chemopreventive agents in patients with familial adenomatous polyposis [3], three randomised trials with similar designs (APPROVe, APC, and PreSAP) were launched between 1999 and 2000. With a five-year follow-up, these studies examined the role of different COXIBs for three years in individuals with a recent history of adenomas. The main objective for all three studies was incidence of adenomas, with the secondary objectives of incidence of advanced adenomas (which included carcinoma in situ and invasive carcinoma) and the number and size of polyps. The three studies, despite being positive for its main objective, were closed early in 2004 after a safety analysis demonstrated an increase in cardiovascular events in some of them, leading to a withdrawal of rofecoxib from the market.

The APPROVe study, which randomised 2587 patients to receive placebo or 25 mg of rofecoxib daily, showed an RR of 0.76 (95% CI 0.69–0.83) for its main objective [20]. The safety analysis of this study initiated the safety analysis of the others, after presenting an RR for cardiovascular events of 1.92 (1.19–3.11). In parallel, the APC trial comparing two doses of celecoxib (200 mg versus 400 mg twice daily) versus placebo showed a reduction for adenoma incidence for the two doses studied (RR 0.67 for 200 mg dose and 0.55 for 400 mg dose) as well as a reduction for advanced adenoma incidence (RR 0.43 and RR 0.34, resp.) [21]. However, a significant increase in the incidence of cardiovascular events was again reported, with an RR of 2.6 and 3.4 for the low and high dose of celecoxib, respectively. After 5 years of follow-up, an update of the data was published in 2009. In it, the chemopreventive effect on adenoma incidence remained with celecoxib use, with an RR of 0.71 for the low dose and 0.62 for the high dose. However, the cardiovascular risk also remained, with an RR of 1.6 (1–2.5) for the 200 mg dose and 1.9 (1.2–3.1) for the 400 mg dose [22]. The last study, PreSAP trial, ran parallel to the APC [23]. It randomised 2 : 1 to receive 400 mg single dose of celecoxib (933 patients) or placebo (628 patients). The RR for the primary endpoint was 0.64, and 0.49 for advanced adenoma incidence; both are significant. This study was also closed in 2004, although the data did not demonstrate an increase in cardiovascular events with an RR of 1.30 (0.65–2.62). Thirty-five patients died due to cardiovascular events or had an episode of heart failure, acute myocardial infarction, or stroke (23 in the experimental group (2.5%) and 12 in the placebo group (1.9%)). A single explanation responsible for the differences between the two trials of celecoxib in the rate of cardiovascular events does not exist, although the difference in dosage and method of administration may explain the differences.

The complete analysis of the cardiotoxicity and cardiovascular event incidence of these trials has been published in several articles (Table 2) [24, 25].

With all these data, in 2007, the USPSTF published a new meta-analysis of the use of NSAIDs and COXIBs in the chemoprevention field and developed its recommendations based on the analysis of the data published in randomised trials, cohorts, and case-control studies [26]. The meta-analysis confirmed the effectiveness of COXIBs in adenoma prevention (RR 0.72, CI 0.68–0.77) and advanced lesion incidence; however, the cardiovascular risk associated with their use prevented the recommendation of their use as chemopreventives except for special groups at high risk of CRC. There are no studies published regarding CRC incidence and mortality with these drugs.

Although there is a lack of comparative studies between ASA, NSAIDs, and COXIBs, COXIBs appear to have a greater effect as chemopreventive agents in CRC. However, the major side effects of COXIBs, mainly cardiovascular, limit their use in healthy individuals.

### 2.3. Non Steroidal Anti-Inflammatory Drugs (NSAIDs)

The data supporting the use of other NSAIDs as chemopreventive agents in CRC come from observational, cohort, and case-control studies. The previously discussed Rostom's meta-analysis described a reduction in CRC incidence of 30–40% and a reduction in adenoma incidence of approximately 45–35% with these drugs [26]. However, they maintain the gastrointestinal toxicity of aspirin, with an ulcer complication rate of 1.5% per year. Other meta-analyses have suggested that a cardiovascular toxicity profile comparable to that of COXIBs is present with NSAID use, especially in relation to dose and duration of treatment [27]. Therefore, the use of NSAIDs as chemopreventive agents currently is not recommended due to the potential toxic side effects.

The combination of different chemopreventive agents is an attractive strategy that would increase the effectiveness of these agents while minimising their side effects. In 2008, a study combining the use of sulindac (NSAID) and difluoromethylornithine (an inhibitor of polyamides synthesis) was published. The study yielded positive results in a population with a recent history of adenomas [28]. An RR of 0.30 (95% CI 0.18–0.49) was obtained for its main objective (adenoma incidence) while a 92% reduction risk in advanced lesions with no significant differences in side effects was also observed. However, an analysis of cardiovascular safety was published a year later, and an increase in cardiovascular events was observed in subjects with cardiovascular risk factors (7 patients in the experimental arm versus 1 patient in the placebo arm) [29]; thus, this strategy, while promising, requires additional studies with additional patients to draw conclusions.

## 3. Familial Adenomatous Polyposis (FAP) Syndrome and CRC

FAP is an autosomal dominant inherited disease caused by mutations in the APC gene on chromosome 5 [30]. It is the most common polyposis syndrome, with a prevalence of 1/10.000, which accounts for approximately 0.5–1% of the total CRC cases. It is characterised by the presence, at an early age, of multiple adenomatous polyps in the colon and rectum (hundreds or thousands), with a cumulative risk of CRC development of nearly 100% in the fourth to fifth decade of life, if not detected and treated early [31]. Prophylactic surgery (pancolectomy with ileal reservoir) is the main strategy for the surveillance and treatment of these patients; however, it does not completely eliminate the risk of developing CRC and does not eliminate the need for an exhaustive follow-up. In addition, there is an associated morbidity with the surgery, and the young age at which the surgery is usually performed has implications on the physical and psychological development of these patients. Therefore, interest has developed regarding chemoprophylaxis that could delay the time of surgery.

In this syndrome, it is difficult to conduct large studies with a large number of patients; thus, scientific evidence is often based on observational and small phase II/III trials. Currently, the drugs with the most evidence as chemopreventive agents in FAP patients are sulindac (NSAIDs) and COXIBs.

### 3.1. Sulindac

In the 1980s, the first case reports demonstrating nearly complete regression of adenomatous polyps in families with FAP treated with sulindac were published [32, 33]. Based on these reports, the first randomised trial of sulindac versus placebo was published in 1991 [34]. In the trial, 20 patients, all with prophylactic surgery and persistence of the rectum, were randomised to receive either sulindac 300 mg daily (10 patients) or placebo (10 patients) for two months. A reduction in rectal polyp incidence (*P* < 0.01) was observed, with complete regression in 6 patients of the experimental arm. The following studies were conducted on populations without prior prophylactic surgery. Two studies conducted by the same research group, Giardiello et al., were published in 1993 and 2002 [35, 36]. With determination of the number of polyps as the main objective, the two studies showed different results. In the first report, 22 patients (18 of them without prophylactic surgery) were randomised to receive either 150 mg of sulindac twice daily or placebo for 9 months. Although there were no significant differences in the demographic characteristics of the two arms, the placebo group had a greater number of polyps at baseline (53 versus 28). After 9 months of treatment, there was a reduction in the number (44%, *P* = 0.014) and size of polyps (35%, *P* < 0.001). These effects could be observed after three months of treatment with a maximum affect after six months but was not maintained after completion of treatment. The rate of side effects was similar in both arms. The second study, published by the same group in 2002, was negative. Forty-one patients with PAF, but without phenotypic expression, were randomised to receive 75 mg or 150 mg of sulindac twice daily (according to body weight) or placebo for 48 months. In addition, rectal biopsies were performed to determine the level of prostaglandins. No significant differences in the number or size of polyps between the two arms were observed, although a significant decrease in prostaglandin levels was reached in the group treated with sulindac. The treatment was well tolerated, with no significant differences in side effects.

Data from additional cohort studies and small, randomised trials have reproduced similar results [37]. While treatment with sulindac appears to induce regression in the number and size of polyps, the effect appears to be restricted to the duration of treatment. There are insufficient data available regarding the use of sulindac or other NSAIDs for chemoprevention in the long term, and there are no data on CRC incidence and mortality; thus, general recommendations cannot be made for using sulindac in patients with FAP at this time.

### 3.2. COXIBs

After the data of animal models showing the role of COXIBs in chemoprevention [37], Steinbach et al. published a phase III trial testing a COXIB, celecoxib, as a chemoprophylactic agent in patients with FAP [38]. Seventy-seven patients, without surgery and with polyps at the baseline colonoscopy, were randomised 2 : 2 : 1 to receive either 100 mg or 400 mg of celecoxib twice daily or placebo for six months. In the demographic characteristics, patients in the 800 mg arm were significantly younger (33.1 years versus 38.6 years, 39.9 years in the placebo arm, *P* = 0.04). The study was positive for the 800 mg dose with a 28% reduction in the number of polyps (*P* = 0.003) and a 30.7% reduction in polyp burden (*P* = 0.001). In global terms, treatment was well tolerated, with diarrhoea and abdominal pain as the most frequent side effects, without significant differences with the placebo arm. These results led to the approval of celecoxib by the FDA as a chemopreventive agent in families with FAP.

After the Steinbach study, additional COXIBs were studied. In 2003, two studies testing 25 mg of rofecoxib were published by both Higuchi et al. [39] and Hallak et al. [40]. Although these studies were small, with 21 and 8 patients enrolled in each, respectively, both included patients who had undergone colectomy (Higuchi, *n* = 13; Hallak, *n* = 5). Both studies confirmed the findings of Steinbach, with a significant reduction in the number and size of the polyps. The treatment was well tolerated, and the most common side effects were diarrhoea and abdominal pain with no significant differences. Interestingly, the Hallak study was the first trial with treatment beyond one year (median 16 months) showing maintenance of the chemopreventive effect with an acceptable clinical tolerance. However, rofecoxib was withdrawn from the market in 2004 after cardiovascular toxicity was reported in other studies of chemoprevention (as described previously in this review).

The current development of COXIBs in the chemoprevention field depends on their cardiovascular safety profile.

### 3.3. Other Drugs

Few additional agents have been studied with success in PAF. However, one study has recently been published, but it was unable to demonstrate an effect. In the Concerted Action Polyp Prevention (CAPP) study, young individuals with APC mutations were randomised using a 2 × 2 factorial design comparing 30 g of resistant starch and 600 mg of aspirin versus placebo for 1 to 12 years [41]. In this study, 206 patients were enrolled, with only 133 used in the analysis of the data. The study was negative for all arms for its main objective (number of adenomas in rectum and sigmoid colon) and for its secondary endpoint (size of adenomas), with no reported side effects of interest.

## 4. Lynch Syndrome and CRC

Lynch syndrome (LS), also called hereditary nonpolyposis colorectal cancer (HNPCC), predisposes individuals at a young age to the development of CRC and other tumours [42]. The germline mutations that produce this syndrome are located in the MMR genes (MLH1, MSH2, MSH6, PMS2). MLH1 mutations are present in 50% of cases, and MSH2 mutations are present in 40% of cases [43].

Microsatellites are susceptible to DNA replication errors when the MMR system is not working correctly. However, 15% of sporadic CRCs can present microsatellites instability (MSI) without a germline mutation in MMR genes; this is related to the somatic hypermetilation of the MLH1 promoter.

CRCs in LS have specific characteristics, such as early age at diagnosis, multiplicity, and family history [44].

Clinical management of CRC consists of surveillance with colonoscopies; additionally, the rarer possibility of prophylactic surgical evaluation exists. The periodic surveillance programs have reduced the CRC mortality in this population approximately 60% [45].

Chemoprevention is currently under investigation in CRC prevention. The molecular understanding of LS leads to hypotheses regarding the reduction of the incidence of CRC in this group. The close relationship between MMR genes and inflammatory pathways has led to the development of clinical trials with different inhibitors in LS.

### 4.1. Aspirin

In preclinical models, aspirin suppressed mutation phenotypes and increased MMR protein expression in MMR-deficient cell lines [46, 47]. In sporadic CRC populations, there is evidence that aspirin leads to a reduction of adenomas. Specifically, in LS, the CAPP2 study analysed the role of aspirin versus placebo (plus starch) in MMR germline mutation carriers. Initially, the trial did not demonstrate a benefit for aspirin; however, 5 years after randomisation, differences were observed between the placebo and aspirin groups. Furthermore, 9 years after randomisation, the aspirin group showed a 50% reduction in CRC incidence, and this effect was stronger in those who received aspirin over 2 years [48].

These results have lead to the evaluation of other drugs, such as celecoxib, that stimulate the TGF-beta signalling pathway. Some studies have shown that specific COX-2 inhibitors may have a higher impact in reducing the number of polyps in mice in contrast with placebo or sulindac [49].

### 4.2. Sulindac

Sulindac, a well-known chemopreventive drug used in adenomatous familial polyposis (AFP), inhibited tumour growth in APC mutant mice but increased tumour appearance in MLH1 mutant mice. Furthermore, additional studies have found differences in adenoma control in relation to the size and location of the adenomas.

## 5. Conclusion

After reviewing the available data from randomised clinical trials and meta-analysis, it is difficult to draw conclusions regarding the effectiveness of NSAIDs and COXIBs in CRC chemoprevention. Both ASA and COXIBs reduce adenoma incidence and, perhaps, they could have an effect on CRC development; however there is lack of comparative trials between these agents. Thus, their impact in CRC mortality is unknown. Dose and duration of treatment remind to be determined and their safety profile may limit their use, mainly in healthy individuals. While promising, more long-term trials are needed in order to set the role of these agents in this field while other areas such as Lynch syndrome need further investigations. Currently, there is only one drug, celecoxib, approved by the regulatory agencies for chemoprevention, in patients with PAF. Inspite of the fact that there is no chemopreventive strategy able to replace surgery and endoscopic surveillance in these patients, it can be seen as an option in selected cases to delay the time of surgery or as secondary prevention if there is persistence of adenomas after prophylactic surgery.

## Figures and Tables

**Table 1:** Results of colorectal cancer and adenoma incidence in aspirin trials.

Study	Year	Cohort	N° cases	Intervention	End Point	RR
PHS (Gann)	1993	Healthy	22071	325 mg every other day versus placebo	CCR incidence	1.15 0.80–1.65
PHS (Stürmer)	1998	Healthy	22071	325 mg every other day versus placebo	CCR incidence	1.03 0.83–1.28
Cook et al.	2004	Healthy	39876	100 mg every other day	CCR incidence	0.97 0.77–1.24
Baron et al.	2003	Prior adenoma	1121	81 mg versus 325 mg daily versus placebo	Adenomas incidence	0.81* 0.69–0.96
Sandler et al.	2003	Prior CCR	635	325 mg daily versus placebo	Adenomas incidence	0.65 0.46–0.91
APPAC	2003	Prior adenoma	272	160 mg versus 325 mg versus placebo**	Adenomas incidence	0.73 0.52–1.04

*Positive for 81 mg arm. **Negative for both arms.

**Table 2:** Results in adenoma incidence in COXIB trials.

Study	Year	Cohort	N° cases	Intervention	End point	RR
APPROVe	2006	Prior adenoma	2587	25 mg rofecoxib versus placebo	Adenoma incidence	0.76 0.69–0.83
APC	2006	Prior adenoma	2035	200 mg bid versus 400 mg bid versus placebo*	Adenoma incidence	0.67 0.55
PreSAP	2006	Prior adenoma	1561	400 mg once versus placebo	Adenoma incidence	0.64 0.56–0.75

*Positive for both arms.
